# Enhancing Histopathological Image Classification Performance through Synthetic Data Generation with Generative Adversarial Networks

**DOI:** 10.3390/s24123777

**Published:** 2024-06-11

**Authors:** Jose L. Ruiz-Casado, Miguel A. Molina-Cabello, Rafael M. Luque-Baena

**Affiliations:** 1 ITIS Software, University of Málaga, C/ Arquitecto Francisco Peñalosa, 18, 29010 Malaga, Spain; joseruizcasado02@gmail.com (J.L.R.-C.); miguelangel@lcc.uma.es (M.A.M.-C.); 2 Instituto de Investigación Biomédica de Málaga y Plataforma en Nanomedicina-IBIMA Plataforma BIONAND, Avenida Severo Ochoa, 35, 29590 Malaga, Spain

**Keywords:** generative adversarial networks, data augmentation, classification, histopathological images, breast cancer

## Abstract

Breast cancer is the second most common cancer worldwide, primarily affecting women, while histopathological image analysis is one of the possibile methods used to determine tumor malignancy. Regarding image analysis, the application of deep learning has become increasingly prevalent in recent years. However, a significant issue is the unbalanced nature of available datasets, with some classes having more images than others, which may impact the performance of the models due to poorer generalizability. A possible strategy to avoid this problem is downsampling the class with the most images to create a balanced dataset. Nevertheless, this approach is not recommended for small datasets as it can lead to poor model performance. Instead, techniques such as data augmentation are traditionally used to address this issue. These techniques apply simple transformations such as translation or rotation to the images to increase variability in the dataset. Another possibility is using generative adversarial networks (GANs), which can generate images from a relatively small training set. This work aims to enhance model performance in classifying histopathological images by applying data augmentation using GANs instead of traditional techniques.

## 1. Introduction

Early detection of breast cancer represents a crucial strategy for reducing mortality rates. Screening methods, such as mammography and ultrasound, are commonly employed for this purpose. Despite the efficacy of these methods in identifying potential abnormalities, determining the malignancy level of a tumor accurately requires the analysis of histopathological images [1]. This process involves digitization of tissue samples obtained through biopsy, which is an invasive procedure. Manual analysis of histopathological images presents several limitations, including laboriousness and a time-consuming nature, in addition to the potential for human error. To address these challenges, computer-aided diagnosis (CAD) [2] systems have been developed to assist healthcare professionals in the decision-making process, providing more accurate and efficient diagnoses.

Technological advances and the development of informatics applications and tools tailored specifically for the processing and analysis of histopathology images have been catalyzed by advancements in technology. These tools have undergone considerable evolution, with modern iterations incorporating cutting-edge deep learning models, including convolutional neural networks (CNNs), to enhance their capabilities for image analysis [3]. CNNs are a class of deep neural networks particularly well-suited for image processing tasks due to their ability to automatically and adaptively learn spatial hierarchies of features through backpropagation. However, for these deep learning neural networks to function successfully, there is a necessity for access to sufficiently large datasets. These datasets are crucial for the training of the neural networks to identify and classify the key features of each class represented in the dataset accurately, and therefore, they must be sufficiently representative.

The acquisition of such datasets presents a significant challenge, primarily due to the high costs and potential risks associated with the procedures used to obtain histopathological images. Furthermore, there is often an inherent imbalance in these datasets, with negative classes being overrepresented compared with positive cases. Addressing these challenges is essential to ensure the effectiveness and reliability of deep learning models in assisting healthcare professionals with breast cancer diagnosis and treatment planning [4].

The assembly of a comprehensive dataset of breast cancer images represents a crucial step in advancing research and diagnosis in the field. However, the scarcity and imbalance of such datasets pose significant obstacles, hindering the development of accurate and robust machine learning models for breast cancer detection and classification. In this context, generative adversarial networks (GANs) emerge as a powerful tool to overcome dataset limitations by generating synthetic images that complement the available data [5]. By capitalizing on the capacity of GANs to generate realistic and diverse images, researchers can enhance existing datasets, balance class distributions, and address gaps in data coverage. This symbiotic relationship between dataset curation and GAN-based augmentation not only enhances the quality and representativeness of the dataset but also strengthens the performance and generalization capabilities of machine learning models trained on it. Consequently, the integration of GANs into the dataset preparation process represents a promising avenue for addressing the data scarcity inherent in breast cancer imaging datasets and advancing the state of the art in breast cancer detection and diagnosis.

Since the introduction of GANs in the seminal work by Goodfellow et al. [6], this architectural paradigm has been widely applied across diverse domains. GANs operate on the principle of employing two distinct models—the generator and the discriminator—engaged in a competitive interplay akin to a zero-sum game. The training of GANs involves fostering competition between two components, namely the generator and the discriminator, which are set up in opposition to one another.

To address the challenges of stability and variability encountered during GAN training, Tero Karras proposed a significant evolution in the network’s architecture [7]. The fundamental concept introduced involves incrementally enhancing the quality of the generated images, referred to as depth. This depth is a hyperparameter that indicates the complexity and resolution of the images the model can generate. During training, ProGAN progressively increases the depth and resolution of the generator and discriminator networks. Initially, the networks commence with a low resolution, simplifying the training process. New layers are then gradually added to both the generator and the discriminator, allowing them to handle higher-resolution images. To circumvent the issue of the generator becoming ensnared by overly similar examples, Karras et al. proposed a strategy involving calculation of the standard deviation across all pixels within the generated mini-batch. This approach enables the discriminator to discern a generated image based on the pixel-level standard deviation present in the batch. This method, known as mini-batch standard deviation, helps maintain diversity in the generated samples and stabilizes the training process, making ProGAN particularly effective for generating high-quality images.

Spectral normalization generative adversarial networks (SN-GANs) [8] represent a variant of generative adversarial networks (GANs) designed to enhance training stability and improve the quality of generated images. This model addresses the problem of Lipschitz continuity, which is crucial for ensuring convergence and preventing mode collapse in GAN training. The SN-GAN achieves this by applying spectral normalization to the weights of the discriminator network, effectively bounding the Lipschitz constant of the discriminator’s mapping function. This normalization technique stabilizes the training process by controlling the Lipschitz norm of the discriminator, thereby mitigating the risk of exploding gradients and facilitating smoother optimization. Consequently, SN-GANs are capable of generating high-quality and diverse synthetic images across a range of datasets, making them a valuable tool in the field of generative modeling.

The boundary equilibrium generative adversarial network (BEGAN) [9] represents an original approach to the GAN structure introduced by Berthelot et al. BEGANs seek to overcome the challenge of training stability and image quality by introducing a novel loss function inspired by the concept of equilibria in game theory. In contrast to traditional GANs, which rely on adversarial loss to guide the training process, BEGANs incorporate an additional measure known as the reconstruction loss. This loss is computed by comparing the original input images with their reconstructions generated by the generator network. BEGANs strive to achieve a balance between the adversarial and reconstruction losses, seeking an equilibrium point where the discriminator cannot distinguish between real and generated images, while the generator can faithfully reconstruct the input distribution. By maintaining this equilibrium, BEGANs produce high-quality and visually coherent images, exhibiting improved training stability and mitigating issues such as mode collapse. This innovative architecture has attracted attention for its ability to generate diverse and realistic images across various datasets, contributing to advancements in the field of generative modeling. In order to prevent model collapse, the model employs techniques proposed in “Energy-based generative network” (EBGAN) and “Advances in Neural Information Processing Systems” [10].

The Re-GAN [11] addresses the challenge of training high-fidelity GANs with limited data. It is designed to alleviate the need for extensive training datasets typically required for such tasks. This method introduces a dynamic architectural reconfiguration process during training, applying pruning to configurations that are not promising [12]. This enables the exploration of diverse subnetwork structures within GANs. The Re-GAN achieves this by iteratively pruning and regrowing connections, which optimizes the network complexity of GANs. This approach stabilizes GAN models, offering an alternative to traditional GAN training methods and the costly process of finding GAN tickets. Furthermore, the versatility of the Re-GAN is demonstrated through its capacity to achieve stability across datasets of varying sizes, domains, and resolutions. Additionally, it is able to function effectively with different GAN architectures, thus providing a data-efficient alternative to other GAN training methods. Furthermore, the Re-GAN enhances performance when combined with recent augmentation techniques, all while requiring fewer floating-point operations and less training time. Notably, the Re-GAN surpasses the state-of-the-art StyleGAN2 without the need for additional fine-tuning steps, thereby demonstrating its effectiveness in improving GAN training efficiency and output quality.

To evaluate the performance of generative networks accurately, it is essential to utilize specific loss functions that are tailored to their unique characteristics. The Wasserstein loss function, as introduced by Arjovsky et al. in their seminal work “Wasserstein Generative Adversarial Networks” [13], leverages the Wasserstein distance to gauge the quality of generated images, thereby enhancing training stability. It is of paramount importance to adhere to the Lipschitz constraint in order for this loss function to work properly. When a gradient penalty is incorporated to enforce compliance with this constraint, the resulting approach is commonly referred to as the Wasserstein loss function with a gradient penalty [14].

To effectively assess a generative model’s performance, it is of paramount importance to conduct a comparison between the generated images and a set of exemplar images. This comparison necessitates a degree of quantifiability, which is essential in enabling meaningful comparisons between different generative models, thus facilitating the identification of the most superior performing model. Among the various metrics that have been introduced in this field, the Frechet inception distance (FID), which was first proposed as an objective standard for evaluating generative models by Heusel et al., has been proven to be a particularly valuable tool in this regard (Heusel et al., cited in [15]). The use of a dataset that differs from ImageNET [16], which is commonly used as a benchmark, results in higher FID values due to differences in feature vector representations [17,18]. This occurs because of the divergence in image content, style, and distribution between datasets, which amplifies the perceptual dissimilarity captured by the FID metric.

**Figure 1:**
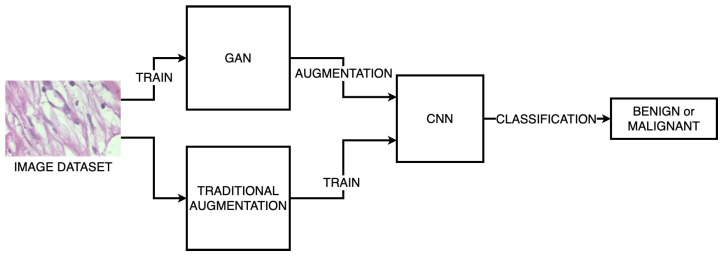
Graphical abstract for conducted experiments.

The primary objective of this paper is to investigate the effectiveness of GAN-based data augmentation compared to traditional augmentation methods in improving the performance of histopathological image classification models. This is illustrated in Figure 1. By systematically comparing the outcomes of these approaches, we aim to discern the advantages and limitations of GAN-based augmentation techniques. Furthermore, we seek to extrapolate our findings to broader contexts within artificial intelligence and generative modeling, providing insights that can guide future investigations in related domains.

## 2. Methods

This section outlines the methodology and procedure used to carry out the experiments. This section presents the methodology and procedural steps employed in the conducted experiments. Multiple GANs were trained to be utilized in image enhancement applications for the dataset. Image enhancement encompasses the addition of supplementary images to the original dataset. Typically, scalar transformations are employed to enhance the variability of the dataset. The trained GANs are utilized to investigate the impact of these networks on the augmentation of images.

In order to accurately assess the impact of generative networks, multiple convolutional models will be utilized while training on the primary image set and on the sets generated with these generative networks. The selected convolutional networks for this evaluation were VGG16, ResNet, and Inception. The selection of these architectures for comparison in the experiments was based on their current status as state-of-the-art convolutional network architectures, being widely recognized for their efficacy in image recognition tasks. These networks represent distinct approaches to feature extraction and representation learning, allowing for comprehensive evaluation across different architectural paradigms. Additionally, their availability as pretrained models with well-established performance benchmarks facilitated fair and rigorous comparison within the scope of the experiments.

The experiments were then divided into two categories: GAN training and CNN training. For each category, a set of configurations and metrics would be established to enable the results of each experiment to be compared.

Regarding the dataset to be used, it was provided by Laboratório Visão Robótica e Imagem UFPR [19]. The images were 700 × 460 and in color, which means they had 3 channels. The images were divided into two classes:

- Benign (With 588 images);
- Malignant (1233 images).

**Figure 2:**
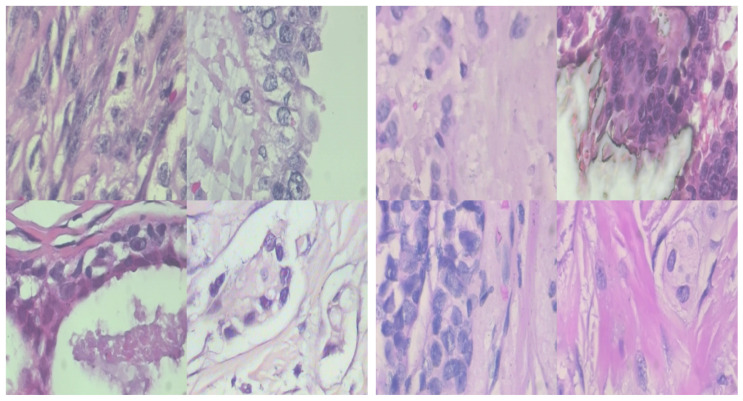
Samples of benign class (**left side**) and malignant class (**right side**).

Figure 2 presents a visual representation of samples from the benign class on the left and the malignant class on the right. This illustration provides a clear visual comparison of histopathological images representing different classes of breast cancer. The benign images exhibit a lighter and more uniform coloration, with soft shades of pink and light purple which collectively give the impression of a consistent and orderly appearance. In contrast, the malignant images are darker and more varied in color with deeper purples and pinks, creating a more intense and uneven appearance. This visual differentiation serves to highlight the overall healthier appearance of the benign tissues in comparison with the more irregular and intense appearance of the malignant tissues.

### 2.1. GAN Training

The issue with the medical imaging set in use is that there was a greater number of malignant images than benign images. In order to address this imbalance in the dataset, several GAN architectures would be trained in an effort to identify the model capable of achieving superior results.

In order to facilitate a comparative analysis of the various GAN architectures, the FID metric would be employed. It should be noted that this metric was not expected to exhibit a specific range of values, given that it is highly dependent on the pretrained weights utilized and the nature of the images employed. Consequently, the FID value for each architecture could be compared with those of the other architectures, with the lowest value representing the optimal performance. Furthermore, a visual comparison of the generated images would be conducted in order to ascertain the degree of similarity between them and the original images. To compute the metric, the package PyTorch FID [20] was used.

The optimizer selected for training the GANs was Adam. This decision was based on its effectiveness in addressing the unique challenges posed by GAN optimization. Adam is distinguished by its adaptive learning rate capabilities, which enable it to dynamically adjust the learning rate for each parameter based on past gradients and moment estimates. This adaptive behavior is particularly beneficial for GAN training, where the generator and discriminator networks often have different learning dynamics and can benefit from different learning rates. Furthermore, Adam’s momentum and second-order moment estimation mechanisms assist in the mitigation of issues such as vanishing or exploding gradients, which are prevalent in GAN training due to the nonlinear and highly volatile nature of the optimization landscape. In summary, Adam’s adaptive learning rate, momentum, and moment estimation features render it an optimal optimizer for GAN training, enabling more stable and efficient convergence while reducing the necessity for manual tuning of the learning rates.

#### 2.1.1. ProGAN

A robust set-balancing system could be achieved by training two generative models—one for each class—using the ProGAN architecture. It is important to note that both models were trained with the same hyperparameter settings. To determine the size of the generated images, the depth value *d* had to be selected to calculate the output size as $2^{\left(d+1\right)}$. Considering the dimensions of the generated images, a depth value of 7 was chosen to ensure that the resulting images were 256 × 256 pixels in size.

An appropriate batch size had to be selected for each depth level, taking into account the output size. It was essential that the size of the selected image matched the output size at each level to prevent erroneous operations in the discriminator and ensure model consistency. The selected values for each depth level were as follows. For depths 1 and 2, the batch size was 64; for depth 3, it was 32; for depth 4, it was 16; for depth 5, it was 4; for depth 6, it was 2; and for depth 7, it was 1. Correspondingly, the image sizes for each depth level were 4, 8, 16, 32, 64, 128, and 256 pixels, respectively.

When a new block was added as the depth level changed, it was not added directly but rather faded in with the previous block. The degree to which the new block faded in was determined by a fade percentage variable, indicating the percentage of the new block utilized in the output. This variable was set to 0.5, establishing a baseline for the degree of fade-in. An external parameter that facilitated accelerated training was the number of threads used for loading the training set. In this instance, four loading threads were defined.

#### 2.1.2. BEGAN

This section examines the configuration of boundary equilibrium generative adversarial networks (BEGANs) with the specific aim of addressing the imbalance inherent in image datasets. The BEGAN, renowned for its ability to generate high-quality and diverse images while maintaining training stability, presents a promising solution for dataset-balancing tasks. By harnessing the equilibrium between generator and discriminator losses, the BEGAN offers a principled framework for generating synthetic images that effectively supplement the existing dataset, thereby promoting class balance and enhancing the robustness of machine learning models. This section explores the key considerations and methodologies involved in configuring the BEGAN for dataset balancing, offering insights into its application in the realm of image dataset management and augmentation.

A total of 100 epochs were utilized for the training process, providing sufficient iterations for the model to learn and refine its parameters. Each epoch involved the processing of data in batches, with a batch size of 64 samples per iteration, ensuring a balance between computational efficiency and model stability. Additionally, a latent dimension of 128 was chosen, determining the dimensionality of the input noise vector that served as the basis for generating synthetic images. This configuration ensured that the model explored a sufficiently rich and diverse latent space, thereby facilitating the generation of high-quality and varied images throughout the training process.

The learning rate of 0.0002 struck a balance between rapid convergence and stability, preventing the model from oscillating or diverging during training. Additionally, the choice of parameters b1 and b2 (0.9 and 0.999, respectively) controlled the exponential decay rates of the first and second moments of the gradients, influencing the optimizer’s behavior. These values were selected to ensure smooth and consistent optimization, allowing the model to effectively learn the intricate dynamics of the data distribution and maintain equilibrium between the generator and discriminator networks throughout training. Overall, the Adam optimizer, with its tailored hyperparameters, provides an effective framework for training BEGAN models, facilitating the generation of high-quality images while promoting training stability and convergence.

#### 2.1.3. SNGAN

In accordance with the previously outlined training configurations, a total of 500 epochs were selected to provide sufficient iterations for the model to learn and refine its parameters. Furthermore, the selection of a latent vector size of 512 contributed to the model’s expressive capacity, enabling it to capture a more diverse range of latent representations. This configuration struck a balance between model complexity and computational efficiency, facilitating robust and high-quality image generation across various datasets and tasks. In summary, the simplicity and effectiveness of the SNGAN configuration demonstrate its versatility and efficacy in the field of generative modeling.

#### 2.1.4. REGAN

In order to assess the effectiveness of the ReGAN across different generative modeling paradigms, the chosen architectures were the ProGAN and SNGAN. These architectures were selected as they possess distinct characteristics and capabilities that would enable the assessment to be carried out. In alignment with the established training protocols for the ProGAN and SNGAN, the same configuration parameters were employed during ReGAN training. This ensures consistency and comparability across experiments. The objective of this study was to elucidate the impact of the ReGAN within the frameworks of the ProGAN and SNGAN with identical configurations for training dynamics, convergence, and the quality of generated outputs. This would provide valuable insights into the effectiveness of the ReGAN across different GAN architectures.

### 2.2. CNN Training

This section of experiments is a comprehensive analysis of convolutional neural network (CNN) training using various datasets and augmentation techniques. The objective was to evaluate the impact of dataset augmentation, traditional transformations, downsampling, and dataset balancing with four different GAN architectures (the ProGAN, SNGAN, BEGAN, and ReGAN) on the performance of CNN models. Three different CNN architectures, namely VGG16, Inception, and ResNet, were evaluated in this experiment. These networks have been proven to be effective in image classification tasks. To investigate the impact of dataset augmentation and GAN-based balancing on CNN performance, we trained these architectures on three datasets: an original dataset, an augmented dataset using traditional transformations, and a balanced dataset generated with each GAN. The objective of this experimental endeavor was to provide valuable insights into the efficacy of data augmentation and GAN-based data balancing techniques in enhancing the performance and robustness of CNNs. This in turn would contribute to advancements in image classification and machine learning research.

In order to ensure the robustness and reliability of the performance metrics, the KFold cross-validation technique was employed to partition the training dataset consistently at each time point throughout the experimentation process. With a choice of k=5, the dataset was divided into five equally sized folds, with each fold serving as a validation set, while the remaining folds were utilized for training. This iterative process was repeated five times, with each fold taking a turn as the validation set. By systematically rotating the dataset partitions, KFold cross-validation minimized the risk of overfitting and provided a more accurate estimation of model performance across different subsets of the data. This approach enhanced the generalization capabilities of the trained models, ensuring that the reported performance metrics were representative and reliable. Through the meticulous application of KFold cross-validation, comprehensive insights into the robustness and effectiveness of the CNN models trained on various datasets and augmented with different techniques were obtained.

Given the limited number of hyperparameters inherent in these networks, their configurations were the same to avoid bias in the results. Therefore, all networks were trained for the full 500 epochs to ensure thorough learning and optimization. In addition, a uniform batch size of 32 was used across all experiments to maintain consistency and comparability in the training process. By standardizing these key training parameters, we aimed to eliminate potential sources of variation and ensure the reliability and fairness of experimental results across different CNN architectures and datasets.

Cross-entropy was chosen as the loss function because of its effectiveness in classification tasks. The cross-entropy loss measures the discrepancy between the predicted class probabilities and the true class labels, penalizing the model for incorrect predictions while encouraging accurate classification. This loss function is well suited for training convolutional neural networks (CNNs) as it efficiently captures the model’s ability to discriminate between different classes and provides meaningful gradients for optimization. By minimizing the cross-entropy loss during training, CNNs are encouraged to learn discriminative features and make more accurate predictions on unseen data. This choice for the loss function was consistent with the goal of achieving optimal classification performance and underscored the emphasis on accuracy and generalization in the experimental set-up.

In addition to selecting cross-entropy as the loss function, the Adam optimizer was chosen to facilitate the training process. In this configuration, Adam operated at a learning rate of 0.0002, which balanced fast convergence with stability, allowing for smooth and efficient training. In addition, the choice of betas, set at 0.5 and 0.999, determined the exponential decay rates for the first and second moments of the gradient, respectively. By adjusting these parameters, Adam optimized the balance between exploration and exploitation during training, allowing the CNNs to effectively navigate the optimization landscape and converge to an optimal solution. This configuration of the Adam optimizer underscores the commitment to achieving robust and reliable results in the experimental set-up and highlights the importance of optimization techniques in training deep learning models for image classification tasks.

## 3. Results

This section presents the outcomes of two distinct experiments conducted to evaluate the efficacy of GAN training and CNN training with balanced datasets. The first experiment focused on utilizing GANs, specifically the ProGAN, SNGAN, BEGAN, and ReGAN, to balance the dataset. This was carried out with the goal of mitigating class imbalance and enhancing the diversity of the training data. The impact of GAN-based dataset balancing techniques on the performance of CNNs trained on these balanced datasets was examined in this experiment. In the second experiment, CNNs were trained directly on the balanced dataset without GAN augmentation, serving as a benchmark to compare against the GAN-trained models.The non-GAN-based techniques employed were random transformations of the images and downsampling of the malignant class to achieve an equal number of images in both classes. By contrasting the outcomes of these two approaches, insights were gained into the effectiveness of GAN-based dataset balancing in improving CNN performance and robustness in image classification tasks.

### 3.1. GAN Training

In order to conduct a comprehensive comparison of the various GAN architectures, the FID metric was employed. The values for the FID relied on the pretrained weights of the Inception network, as highlighted in [17,18]. It was imperative to utilize the same Inception network across all GAN architectures to ensure consistency and fairness in the comparison process. The utilization of a standardized Inception network ensured uniformity within the evaluation framework, thereby enabling an unbiased assessment of the performance of each GAN architecture. This approach allowed for meaningful comparisons based on the FID metric, facilitating insights into the relative strengths and weaknesses of the different GAN models.

**Figure 3:**
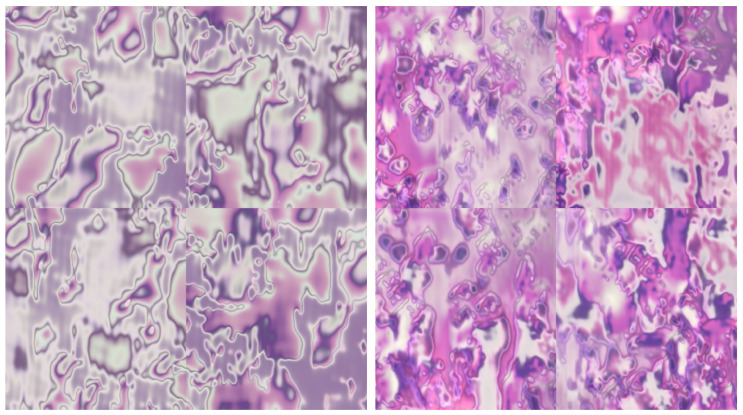
Images generated for benign class (**left**) and malignant class (**right**) with the ProGAN, which was the GAN that achieved the lowest FID value in our experiments.

Upon reviewing the FID results presented in Table 1, it became evident that all models, with the exception of ReGAN SNGAN, exhibited FID values below 400. This suggests relatively favorable performance in terms of image quality and diversity. In particular, ProGAN presented the lowest FID values for the benign and malignant classes, indicating superior performance in generating high-quality images compared with the other models (see Figure 3 for visualizations of images generated for both classes). However, the FID values for ReGAN SNGAN exceed the threshold, indicating potential challenges or limitations in achieving satisfactory quality in image generation. These FID results provide valuable information on the performance of the various models, guiding future analysis and refinement efforts.

GANs demonstrating FID values below 400 were deemed suitable candidates for dataset balancing. This criterion ensured that the chosen GANs exhibited favorable performance in generating high-quality and diverse images, thereby effectively balancing the dataset. Furthermore, it is crucial to highlight that the outputs generated by the selected GANs seamlessly adapted to the input requirements of the CNNs used for image classification. By aligning the characteristics of the GANs’ outputs with the specifications of the CNNs’ inputs, smooth integration is achieved, facilitating the utilization of GAN-generated data to enhance the robustness and performance of the CNN models in image classification tasks. This symbiotic relationship between GANs and CNNs underscores their complementary roles in the dataset balancing and classification processes, ultimately contributing to improved model accuracy and generalization capabilities.

### 3.2. CNN Training

**Figure 4:**
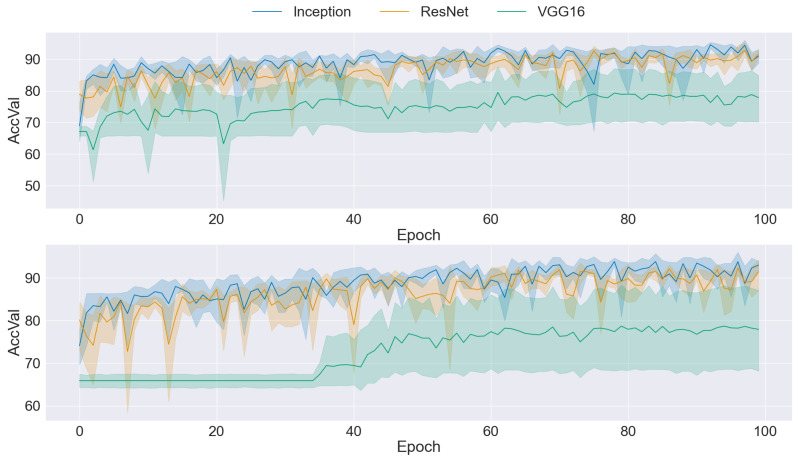
Comparison of training data performance: original dataset (**upper**) vs. traditional augmentation techniques (**lower**).

**Figure 5:**
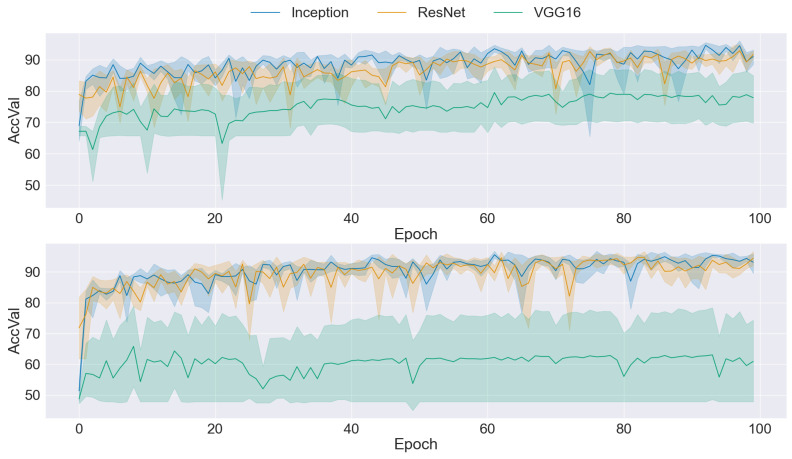
Comparison of training data performance: original dataset (**upper**) vs. downsampling malignant class (**lower**).

**Figure 6:**
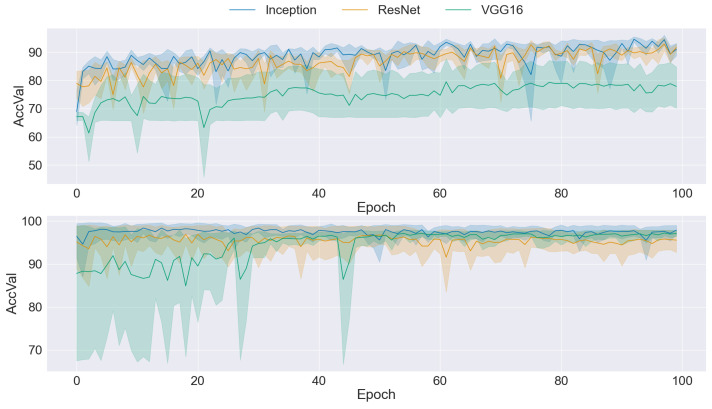
Comparison of training data performance: original dataset (**upper**) vs. BEGAN augmentation (**lower**).

**Figure 7:**
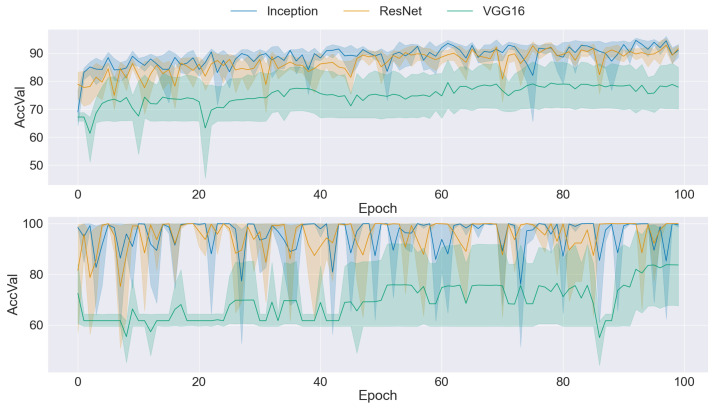
Comparison of training data performance: original dataset (**upper**) vs. ProGAN augmentation (**lower**).

**Figure 8:**
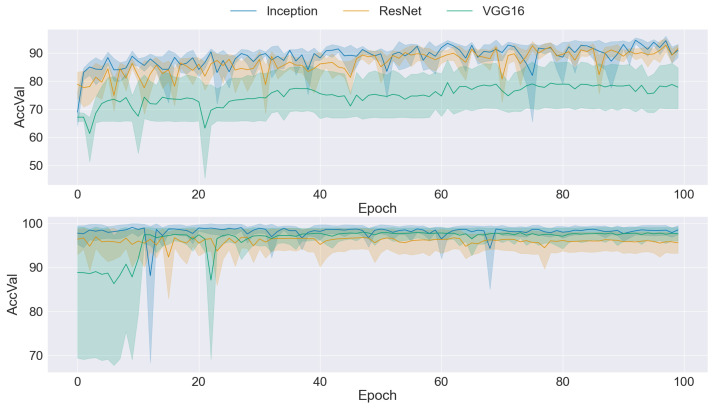
Comparison of training data performance: original dataset (**upper**) vs. ReGAN ProGAN augmentation (**lower**).

The graphs presented in Figure 4, Figure 5, Figure 6, Figure 7 and Figure 8 offer a comprehensive overview of the performance dynamics of CNNs across various epochs and folds in a fivefold cross-validation set-up. Each graph provides insights into the mean accuracy and standard deviation of the CNN models trained using different datasets and methodologies. The upper side graph illustrates the performance metrics derived from training the CNN models on the original dataset. In contrast, the lower side graph showcases the corresponding metrics obtained from each data augmentation technique applied to the dataset (GAN). In both graphs, three distinct CNN architectures (Inception, ResNet, and VGG16) are evaluated, with the mean accuracy and standard deviation depicted for each architecture. The shaded regions surrounding the lines represent the standard deviation, offering insights into the variability in model performance across different folds and epochs. These visualizations provide a comprehensive understanding of the impact of data augmentation techniques on CNN training and performance variability, informing further analysis and optimization efforts in image classification tasks.

A comparison of the two graphs in Figure 4 reveals an improvement in mean accuracy when traditional data augmentation techniques are applied to the dataset. However, despite this enhancement in accuracy, it is evident that the standard deviation remained relatively high across epochs and folds. This observation suggests that while traditional data augmentation contributes to improved overall performance, it may not fully address the variability in model performance across different folds and epochs. Consequently, while the mean accuracy was positively influenced by traditional transformation, the persistently high standard deviation highlights the necessity for further investigation and refinement in training methodologies to achieve greater consistency and stability in CNN performance.

A comparison of the results obtained from the original dataset (upper) with those obtained by applying downsampling to the malignant class to balance the number of images (shown in Figure 5) revealed distinct trends across different CNN architectures. For the Inception and ResNet models, the results were found to be quite similar between the two datasets, with a slightly higher accuracy observed for the downsampled dataset. This indicates that both architectures benefitted marginally from the balanced dataset, likely due to a reduction in class imbalance. However, the results for the VGG16 model were significantly worse when using the downsampled dataset. This suggests that the reduced number of images in the malignant class was insufficient for effective training, indicating that VGG16 was more sensitive to the size of the training dataset and that a larger number of images was necessary to achieve optimal performance.

Figure 6 presents a comparison graph illustrating the training mean accuracy and standard deviation when using BEGAN as the dataset balancer. The graph reveals a notable improvement in mean accuracy across all three CNN architectures, indicating enhanced model performance when trained on the dataset balanced with BEGAN. Additionally, there was an overall reduction in standard deviation, suggesting improved stability and consistency in model performance. Nevertheless, it is notable that the standard deviation remained relatively high for the Inception architecture, indicating variability in performance across different epochs and folds. Despite this exception, the general trend reflects the efficacy of BEGAN in improving both the accuracy and stability of the CNN models, underscoring its potential as a dataset balancing technique in image classification tasks.

Figure 7 presents a comparison between CNNs trained on the original dataset (upper side) and CNNs trained with the ProGAN as the dataset balancer (lower side). While the accuracy demonstrated improvement with ProGAN-balanced datasets, the higher standard deviation indicates a noticeable increase in instability. Despite the ProGAN having the lowest FID among the GAN architectures considered, the results suggest a trade-off between image quality and variability. The CNNs trained with ProGAN-balanced datasets exhibited high-quality images, as reflected by the low FID values. However, they also displayed more significant variability in performance across epochs and folds, as evidenced by the elevated standard deviation. These findings underscore the complex interplay between image quality and stability in CNN training, highlighting the need for further exploration and optimization to balance these competing factors.

Figure 8 presents a comparison between the CNNs trained with the original and ReGAN ProGAN balanced datasets. While the accuracy achieved with ReGAN ProGAN was not as high as that with ProGAN, there was a notable improvement in stability, as evidenced by the reduced variability in accuracy values across epochs and folds. However, despite this improvement in stability, the standard deviation remained high, indicating ongoing variability in model performance. Although ReGAN ProGAN demonstrated a balance between accuracy and stability, the persistence of a high standard deviation indicates that further optimization may be necessary to achieve greater consistency in model performance.

Table 2 presents the mean accuracy and mean loss alongside their respective standard deviations for various CNN models trained with different data augmentation techniques. The table illustrates the impact of distinct augmentation strategies on model performance. Traditional image augmentation demonstrated a modest enhancement in mean accuracy coupled with a reduction in the standard deviation, implying improved consistency in model performance. Conversely, the augmentation techniques involving generative adversarial networks (GANs) exhibited significant improvements in both mean accuracy and standard deviation, indicating enhanced model robustness and stability. It is noteworthy that ReGAN ProGAN emerged as the top performer, exhibiting the highest mean accuracy and the lowest standard deviation among the evaluated methods.

The outcomes of the Inception model indicate that conventional image transformations resulted in a marginal improvement in both accuracy and standard deviation. Conversely, the GAN-based augmentation techniques demonstrated a substantial enhancement in both accuracy and standard deviation, thereby underscoring their efficacy in improving model performance. Among the GAN-based augmentation methods, the ProGAN stood out with the highest accuracy while maintaining a similar standard deviation compared to other techniques.

The ResNet model presented analogous results, corroborating the trends observed for the Inception model. In all cases, the GAN-based augmentation techniques consistently provided the highest average accuracy compared with the original dataset and traditional augmentation methods. It should be noted that although GAN-based augmentation improved the accuracy, it was often accompanied by a slightly higher standard deviation, indicating greater variability in model performance. Nevertheless, the ReGAN ProGAN model (highlighted in the table) exhibited a notable improvement in accuracy, accompanied by a marginal increase in the standard deviation.

The VGG16 model emerged as the worst-performing CNN among the architectural variants considered. Overall, VGG16 had a lower average accuracy and a higher standard deviation compared with the other CNN models. Despite these challenges, it can be observed that remarkable performance improvements were achieved with the ReGAN ProGAN and BEGAN enhancement techniques. Despite the inherent limitations of VGG16, the ReGAN ProGAN (highlighted in the table as the best performer) and, to a lesser extent, BEGAN models demonstrated commendable performance, with higher mean accuracies and relatively lower standard deviations compared with other augmentation methods. These results underscore the resilience and effectiveness of the ReGAN ProGAN and BEGAN models in improving the performance of even the worst-performing CNN architecture.

The results, presented in Table 3, provide an overview of the precision, recall, and F1 score for each CNN and augmentation technique. The metrics were computed by aggregating all true labels and predictions across every test fold, which is why the standard deviations were not included. The data indicate that the ProGAN and ProGAN with ReGAN regularization consistently demonstrated the highest performance across all metrics. This indicates that these GAN-based augmentation techniques significantly enhanced the model’s ability to generalize from the training data. Conversely, the original dataset and traditional augmentation methods exhibited lower precision, indicating a lack of generalization capability and an increased likelihood of false positives. Furthermore, downsampling has been observed to demonstrate improved generalization compared with the original dataset and traditional augmentation methods. However, it still fell short when compared with all the GAN-based techniques. These findings highlight the potential of GAN-based augmentations, particularly the ProGAN and its regularized variant, in enhancing the robustness and accuracy of histopathological image classification models.

In order to highlight the performance of our model, we present the confusion matrix for the Inception CNN, which demonstrated the most favorable results in our study. The confusion matrix serves as an effective visualization tool, illustrating the model’s classification accuracy and its ability to distinguish between benign and malignant histopathological breast cancer images. By focusing on the Inception CNN, we aim to provide a clear representation of its efficacy, emphasising its potential as a robust tool for augmenting histopathological image analysis.

The confusion matrix for the original dataset, presented in Table 4, reveals a relatively low precision in classifying the benign class, as evidenced by a significant number of false positives (12%). Additionally, while the model demonstrated high recall for the malignant class (94%), the overall precision for the benign classifications was compromised, indicating room for improvement in distinguishing between benign and malignant cases more accurately.

The confusion matrix for the dataset with traditional augmentation, as shown in Table 5, indicates that the precision for benign classifications was even lower than that of the original dataset, with a false positive rate of 16%. While there was an improvement in the recall for malignant lesions (96%), the overall precision for benign cases decreased. This suggests that traditional augmentation is not an efficient technique for this problem, as it failed to enhance the model’s ability to accurately differentiate between benign and malignant lesions.

The confusion matrix for the dataset with downsampling, as shown in Table 6, demonstrates a notable improvement over the previous two methods. Both benign and malignant classifications achieved a precision of 94%. This indicates that an equal number of images per class significantly enhanced the model’s performance. Nevertheless, while this balanced approach yielded superior overall results, the metrics, particularly the false positive and false negative rates (both at 5%), indicate that there is still potential for enhancement in the model’s accuracy.

Table 7 presents the confusion matrix for the augmented dataset, which was generated using the BEGAN model. This demonstrates a notable improvement in model performance. The precision for both benign and malignant classifications was exceptionally high, with only 2% false positives for benign lesions and 1% false negatives for malignant lesions. This evidence indicates that the BEGAN-based augmentation technique is highly effective, substantially enhancing the model’s ability to accurately distinguish between benign and malignant histopathological breast cancer images.

The confusion matrix in Table 8 for the augmented dataset, which incorporated the ProGAN-based technique, demonstrates outstanding performance. The model achieved near-perfect precision for both benign and malignant classifications, with only 1% false positives and 1% false negatives. This demonstrates the efficacy of the ProGAN-based augmentation, markedly enhancing the model’s capacity to accurately differentiate between benign and malignant histopathological breast cancer images.

The confusion matrix depicted in Table 9 illustrates the outcomes of the ReGAN ProGAN-based augmentation technique. Notably, the model achieved an impressive level of precision for both benign and malignant classifications, demonstrating only 2% false positives and 2% false negatives. This highlights the efficacy of the ReGAN ProGAN approach in enhancing the model’s capacity to distinguish between benign and malignant histopathological breast cancer images. It also reinforces the utility of this approach in augmenting image datasets, thereby improving classification accuracy.

In conclusion, the results presented in the confusion matrices demonstrate that the ProGAN-based augmentation technique consistently outperformed the other methods, including the ReGAN ProGAN approach. In a series of experiments, the ProGAN demonstrated near-perfect precision for both benign and malignant classifications, with minimal false positives and false negatives. While the ReGAN ProGAN method also demonstrated impressive performance, the ProGAN consistently maintained a slight edge in accuracy. These findings reinforce the robustness and efficacy of the ProGAN in enhancing the classification accuracy of histopathological breast cancer images, thereby reaffirming its status as a leading augmentation technique in this domain.

## 4. Conclusions

The research direction established for this study was to leverage generative adversarial networks (GANs) to enhance breast cancer diagnosis systems based on histopathological images. To this end, a variety of generative architectures (the BEGAN, ProGAN, SNGAN, and ReGAN) were trained and evaluated in order to ascertain their efficacy. Following the training of these generative models, their effectiveness in balancing the existing image datasets was examined. This study specifically investigated the enhancements resulting from incorporating these balanced datasets into the training of state-of-the-art convolutional neural networks (CNNs), including Inception, ResNet, and VGG16.

Upon examination of the FID results, it became evident that the ProGAN attained the lowest value, indicative of its ability to generate highly realistic images. This highlights the capacity of the ProGAN to generate images that closely resemble authentic samples, a pivotal aspect in optimizing the efficacy of breast cancer diagnosis systems. Moreover, both the BEGAN and ReGAN ProGAN models exhibited commendable FID values, which suggests that they are capable of generating images with a high degree of realism. These findings demonstrate the efficacy of the BEGAN and ReGAN ProGAN approaches as viable alternatives for dataset balancing and image generation. They also validated their utility in improving the performance of breast cancer diagnosis systems based on histopathological images.

The outcomes of training the CNNs demonstrated a notable trend; a higher mean accuracy and lower standard deviation (STD) were consistently observed compared with both the original dataset and traditional augmentation methods. Furthermore, the GAN-based augmentation techniques demonstrated enhanced accuracy and precision, as well as improved recall and F scores, thereby reinforcing their potential to enhance diagnostic systems. This overarching trend demonstrates the efficacy of GAN-based augmentation techniques in enhancing the robustness and stability of the trained models. It is notable that the ProGAN, despite exhibiting the lowest FID value, indicative of its ability to generate highly realistic images, exhibited a relatively higher standard deviation. This observation indicates that while the ProGAN was highly effective in generating realistic images, it may not explore as much variability in the generated samples. Nevertheless, the superiority of the GAN-based augmentation techniques was evident across all CNN architectures, including those with worse performance, such as VGG16. In conclusion, the findings demonstrate the efficacy of GAN-based augmentation in improving model performance, indicating its potential to enhance breast cancer diagnosis systems based on histopathological images, regardless of the CNN architecture used.

It is essential to acknowledge that these outcomes are specific to the domain of histopathological image analysis for breast cancer diagnosis. The observed performance enhancements with GAN-based augmentation cannot be directly extrapolated to other domains or applications without further investigation. Each application may present unique challenges and characteristics that could affect the effectiveness of these techniques.

In conclusion, the comparative analysis of GAN-based data augmentation methods (BEGAN, ProGAN, and REGAN ProGAN methods) revealed that the ProGAN achieved the best results in terms of accuracy, precision, recall, and F1 score for augmenting histopathological images. Notably, the ProGAN with ReGAN regularization exhibited similar performance metrics to the ProGAN but with a reduced standard deviation, indicating more consistent results. The architectural advantage of the ProGAN lies in its progressive growing mechanism, which allows the model to incrementally increase the complexity and resolution of generated images. This progressive training strategy helped stabilize the training process and enhance the quality of generated images, making the ProGAN highly effective for medical image augmentation. In contrast, the BEGAN, while useful, does not employ this progressive approach, and thus it fell short in generating images of comparable quality and consistency. The findings of this study indicate that the ProGAN, particularly when augmented with ReGAN regularization, represents a promising approach for enhancing medical image analysis. It is recommended that further investigation of the efficacy of the ProGAN in different imaging domains be conducted.

In our evaluation of different CNNs for histopathological image classification, we found that the Inception network achieved the best overall performance. Inception outperformed the other models in terms of accuracy, precision, recall, and F1 score. Notably, the VGG16 network exhibited a high standard deviation in its results, indicating inconsistent performance. This variability can be attributed to the older architecture of the VGG16 network, which requires a substantial amount of training data to perform optimally and lacks the advanced optimizations present in more recent networks. The ResNet network performed adequately but did not match the superior results of the Inception network. These findings indicate that the Inception network’s architectural innovations, such as the use of inception modules that facilitate more efficient and scalable feature extraction, render it particularly well suited for utilization with generative adversarial network (GAN)-based augmentation tools in medical image classification. Future studies should further examine the potential of Inception in conjunction with GAN-generated data across diverse imaging applications.

## Figures and Tables

**Table 1:** FID values achieved by each GAN architecture. Best result is highlighted in **bold**.

Model	Benign	Malignant
BEGAN	349.09	331.16
**ProGAN**	**261.51**	**222.81**
SNGAN	424.93	401.71
ReGAN ProGAN	372.64	306.53
ReGAN SNGAN	473.95	485.67

**Table 2:** Test results for CNN training. Best values for each model are highlighted in **bold**.

Model	Dataset	Acc	Loss
Inception	Original	92.03 ± 3.64	0.23 ± 0.13
Inception	Traditional Augmentation	92.41 ± 2.81	0.19 ± 0.05
Inception	Downsampling	94.47 ± 2.87	0.14 ± 0.05
Inception	BEGAN	97.97 ± 1.54	0.07 ± 0.06
**Inception**	**ProGAN**	**99.36 ± 2.57**	**0.02 ± 0.04**
Inception	ReGAN ProGAN	98.45 ± 1.46	0.06 ± 0.06
ResNET	Original	92.63 ± 2.28	0.21 ± 0.06
ResNET	Traditional Augmentation	90.98 ± 4.36	0.15 ± 0.10
ResNET	Downsampling	94.89 ± 2.87	0.14 ± 0.05
ResNET	BEGAN	95.97 ± 4.13	0.22 ± 0.26
ResNET	ProGAN	97.26 ± 5.01	0.01 ± 0.14
**ResNET**	**ReGAN ProGAN**	**96.47 ± 2.72**	**0.16 ± 0.16**
VGG16	Original	79.45 ± 10.64	0.47 ± 0.14
VGG16	Traditional Augmentation	78.57 ± 8.79	0.48 ± 0.14
VGG16	Downsampling	61.12 ± 19.65	0.56 ± 0.18
VGG16	BEGAN	96.52 ± 5.43	0.23 ± 0.08
VGG16	ProGAN	85.10 ± 20.21	0.26 ± 0.35
**VGG16**	**ReGAN ProGAN**	**94.47 ± 2.10**	**0.11 ± 0.10**

**Table 3:** Test results for CNN test set. Best values for each model are highlighted in **bold**.

Model	Dataset	Precision	Recall	F-SCORE
Inception	Original	0.87	0.93	0.91
Inception	Traditional Augmentation	0.83	0.96	0.89
Inception	Downsampling	0.94	0.94	0.94
Inception	BEGAN	0.97	0.98	0.97
**Inception**	**ProGAN**	**0.99**	**0.99**	**0.99**
Inception	ReGAN ProGAN	0.98	0.98	0.98
ResNET	Original	0.91	0.93	0.91
ResNET	Traditional Augmentation	0.84	0.93	0.88
ResNET	Downsampling	0.96	0.93	0.94
ResNET	BEGAN	0.95	0.96	0.95
**ResNET**	**ProGAN**	**0.98**	**0.99**	**0.99**
ResNET	ReGAN ProGAN	0.94	0.98	0.96
VGG16	Original	0.44	0.91	0.60
VGG16	Traditional Augmentation	0.40	0.92	0.56
VGG16	Downsampling	0.67	0.59	0.63
VGG16	BEGAN	0.97	0.97	0.97
VGG16	ProGAN	0.97	0.71	0.83
**VGG16**	**ReGAN ProGAN**	**0.98**	**0.98**	**0.98**

**Table 4:** Confusion matrix for original dataset.

Label	Benign	Malignant
Benign	0.87	0.12
Malignant	0.05	0.94

**Table 5:** Confusion matrix for traditional augmentation.

Label	Benign	Malignant
Benign	0.83	0.16
Malignant	0.03	0.96

**Table 6:** Confusion matrix for downsampling.

Label	Benign	Malignant
Benign	0.94	0.05
Malignant	0.05	0.94

**Table 7:** Confusion matrix for BEGAN-based augmentation.

Label	Benign	Malignant
Benign	0.97	0.02
Malignant	0.01	0.98

**Table 8:** Confusion matrix for ProGAN-based augmentation.

Label	Benign	Malignant
Benign	0.99	0.01
Malignant	0.01	0.99

**Table 9:** Confusion matrix for ReGAN ProGAN-based augmentation.

Label	Benign	Malignant
Benign	0.99	0.01
Malignant	0.01	0.99

## Data Availability

The dataset used in this study is openly available [19]. The source code developed for this work is openly available (GitHub Repository) https://github.com/icai-uma/Enhancing-Classification-Performance-through-Synthetic-Data-Generation-with-GANs.
